# Ileus in children presenting with diarrhea and severe acute malnutrition: A chart review

**DOI:** 10.1371/journal.pntd.0005603

**Published:** 2017-05-11

**Authors:** Mohammod Jobayer Chisti, Abu SMSB Shahid, K. M. Shahunja, Pradip Kumar Bardhan, Abu Syeed Golam Faruque, Lubaba Shahrin, Sumon Kumar Das, Dipesh Kumar Barua, Md Iqbal Hossain, Tahmeed Ahmed

**Affiliations:** Nutrition and Clinical Services Division (NCSD), International Centre for Diarrhoeal Disease Research, Bangladesh (icddr,b), Dhaka, Bangladesh; University of California San Diego School of Medicine, UNITED STATES

## Abstract

**Background:**

Severely malnourished children aged under five years requiring hospital admission for diarrheal illness frequently develop ileus during hospitalization with often fatal outcomes. However, there is no data on risk factors and outcome of ileus in such children. We intended to evaluate predictive factors for ileus during hospitalization and their outcomes.

**Methodology/Principal findings:**

This was a retrospective chart review that enrolled severely malnourished children under five years old with diarrhea, admitted to the Dhaka Hospital of the International Centre for Diarrhoeal Disease Research, Bangladesh between April 2011 and August 2012. We used electronic database to have our chart abstraction from previously admitted children in the hospital. The clinical and laboratory characteristics of children with (cases = 45), and without ileus (controls = 261) were compared. Cases were first identified by observation of abnormal bowel sounds on physical examination and confirmed with abdominal radiographs. For this comparison, Chi-square test was used to measure the difference in proportion, Student’s t-test to calculate the difference in mean for normally distributed data and Mann-Whitney test for data that were not normally distributed. Finally, in identifying independent risk factors for ileus, logistical regression analysis was performed. Ileus was defined if a child developed abdominal distension and had hyperactive or sluggish or absent bowel sound and a radiologic evidence of abdominal gas-fluid level during hospitalization. Logistic regression analysis adjusting for potential confounders revealed that the independent risk factors for admission for ileus were reluctance to feed (odds ratio [OR] = 3.22, 95% confidence interval [CI] = 1.24–8.39, p = 0.02), septic shock (OR = 3.62, 95% CI = 1.247–8.95, p<0.01), and hypokalemia (OR = 1.99, 95% CI = 1.03–3.86, p = 0.04). Mortality was significantly higher in cases compared to controls (22% vs. 8%, p<0.01) in univariate analysis; however, in multivariable regression analysis, after adjusting for potential confounders such as septic shock, no association was found between ileus and death (OR = 2.05, 95% CI = 0.68–6.14, p = 0.20). In a separate regression analysis model, after adjusting for potential confounders such as ileus, reluctance to feed, hypokalemia, hypocalcemia, and blood transfusion, septic shock (OR = 168.84, 95% CI = 19.27–1479.17, p<0.01) emerged as the only independent predictor of death in severely malnourished diarrheal children.

**Conclusions/Significance:**

This study suggests that the identification of simple independent admission risk factors for ileus and risk factors for death in hospitalized severely malnourished diarrheal children may prompt clinicians to be more vigilant in managing these conditions, especially in resource-limited settings in order to decrease ileus and ileus-related fatal outcomes in such children.

## Author summary

Childhood malnutrition and diarrhea are important problems in lower and middle-income countries, including Bangladesh. Severe acute malnutrition (SAM) and diarrhea are responsible for more than one third of all deaths in children below five years old globally. Most of these deaths occur because of complications of SAM and/or diarrhea. SAM and diarrhea may simultaneously present in a child, often with serious complications. Ileus is a common fatal complication in such hospitalized children, and is accompanied by abdominal distension and hyperactive, sluggish, or absent bowel sounds heard using a stethoscope. Ileus is confirmed by radiologic evidence of multiple gas-fluid levels in the abdomen. However, the underlying factors contributing to ileus and its management in diarrheal children with severe acute malnutrition is unknown. Our study aimed to ascertain these risk factors and the outcome of ileus in such children by reviewing the data of previously admitted children between April 2011 and August 2012. Of 306 severely malnourished Bangladeshi under-five-year-old children with diarrhea enrolled for 17 months, 45 had ileus. Ileus was independently associated with a reluctance to feed, septic shock, and hypokalemia and had a higher case-fatality rate compared to those without ileus.

## Introduction

Globally, diarrhea accounted for 9% of an estimated 5.9 million deaths in children under five years old in 2015 [1]. Most of the deaths occurred in lower and middle-income countries including Bangladesh [2]. The case-fatality rate (CFR) from diarrhea in Bangladesh was 6% among an estimated 119,000 deaths in children less than five years old [1]. The majority of the deaths occurred due to a number of immediate complications of diarrhea such as dyselectrolytemia, sepsis, and ileus [3]. However, severe acute malnutrition (SAM) was one of the important causes of death in under-five-year-old children and the risk of death from any cause was 9 times higher for SAM compared to non-SAM children [4]. CFR among children hospitalized with SAM has virtually remained unchanged over the past several decades in many centers [5]. Some centers have been able to reduce CFR to less than 5% by minimizing complications from SAM via the implementation of World Health Organization (WHO) guidelines [6]. These complications were life-threatening in children presenting with severe malnutrition and were often associated with death [3]. Ileus, a condition accompanied by abdominal distension and hyperactive, sluggish, or absent bowel sounds and radiologic evidence of multiple gas-fluid levels in the abdomen, is considered as one of the serious ramifications of diarrhea [7]. It is suggested that ileus could be more deleterious to the health of diarrheal children with severe malnutrition compared to those without severe malnutrition.

Ileus was found to be common in critically ill children and adults and most often reflected the severity of underlying disease [8]. Ileus usually presents with distension and tenderness of the abdomen and abnormal bowel sounds, especially in hospitalized children [9]. Recent studies have revealed that a significant proportion of hospitalized children experience ileus due to sepsis, dyselectrolytemia, and pseudo-membranous colitis in malnutrition [10,11]. On the other hand, ileus in adults mostly presents as an acute abdomen characterized by abdominal distension and an ischemic bowel with or without perforation [12]. A previous study from Bangladesh revealed that 12% of children with diarrhea developed ileus and 25% of them died [9]. The prevalence and fatal outcome of ileus is considered to be higher in children with SAM compared to those without SAM. However, WHO has not recommended any management plan for ileus in such children because of a lack of evidence regarding underlying factors that contribute to ileus in children having diarrhea with SAM. Identifying the factors contributing to ileus in children having diarrhea with SAM may help clinicians to deploy early and appropriate intervention that should further help to reduce deaths, especially in developing countries.

In the Dhaka hospital of the International Centre for Diarrhoeal Disease Research, Bangladesh (icddr,b), diarrhea patients with and without severe malnutrition are treated [13], and a substantial number of these patients develop ileus during hospitalization [7]. If such cases are promptly identified and factors associated with ileus in severely malnourished children with diarrhea are treated, it might be possible to prevent the serious complications and death due to ileus. However, to our knowledge, there are no published data on the risk factors and outcome of ileus in children with diarrhea presenting with SAM. Thus, our objective was to identify the factors that predicted ileus and to evaluate the outcome in such children.

## Materials and methods

### Ethics statement

The data used in this study were retrieved from the case records of patients of the Dhaka hospital of icddr,b. Data were entered in an anonymized manner prior to analysis and used for the improvement of the quality of care of the hospital patients. The study was approved by the Ethical Review Committee of icddr,b.

### Study setting

The hospital in this study is an urban diarrheal hospital situated in Dhaka, the capital city of Bangladesh. Annually, approximately 150,000 patients are seen and the presence of diarrhea is compulsory for admission. There are 350 beds in this hospital; however, during diarrheal epidemics, extra beds are often used to treat the additional patients. Two epidemics are commonly observed in this region: one in the hot summer of April-May and another in September-October. More than 90% patients are treated in the Short-Stay Ward (SSW). Those without diarrheal complications or associated problems receive treatment in the SSW and their median duration of stay is 24 hours. Patients with diarrheal complications or associated problems are treated in the Longer-Stay Ward (LSW) or in the Intensive Care Unit (ICU). The median duration of stay at the LSW and ICU is 3 days and 5 days, respectively. A detailed description of Dhaka hospital has been provided elsewhere [14].

### Study design

We employed a retrospective chart analysis with an unmatched case-control design. A matched case-control design was not conducted as there was not enough power for a matched design because of the small sample size. The study enrolled children aged 0–59 months, with severe malnutrition and diarrhea, admitted to the ICU or LSW of the Dhaka ICDDR,B hospital, and with information regarding abdominal examination including ileus as a part of standard care in the ICU between April 2011 and August 2012. The enrolled children with diarrhea who developed ileus during hospitalization constituted our cases, whereas those who did not develop ileus constituted controls. Ileus was defined as the development of abdominal distension with hyperactive, sluggish, or absent bowel sounds and radiologic evidence of abdominal gas-fluid level [7] during hospitalization. Severe malnutrition was defined following WHO anthropometry as described elsewhere [15]. We used the electronic database of the hospital to perform our chart abstraction, from children previously admitted in the hospital. Those involved in the abstraction were not involved in the care of these patients when they were hospitalized. Events in the ICU were stored in an electronic medical record. Socio-demographic history, clinical features, and laboratory investigations reports are automatically collected in this database. First, the database was queried to identify children below 5 years old admitted for diarrhea, and then these charts were reviewed for the diagnosis of severe malnutrition. Regarding the severely malnourished children with diarrhea, the database was further queried to establish whether these children had clinically and radio-graphically confirmed ileus, and the cases and controls were differentiated via the information received. At different time points, we had collected data from more than one individual in the Information and Technology (IT) Department of icddr,b and during the collection of our data we did not find any inconsistency among the datasets.

### Patient management

All the children with ileus received standard conservative medical treatment following hospital guidelines derived from Ahmed et al [3]. No patient required surgical intervention. In managing ileus, food was given at 3-4-hourly instead of the routine 2-hourly intervals. If the condition was not resolved within 6–8 hours of spacing of diets, a single intramuscular injection of magnesium sulphate 50%, 0.3 ml/kg was administered, up to a maximum of 2 ml. This bolus dose was in addition to the daily maintenance dose of magnesium sulphate injection. If symptoms resolution was not achieved within 2 hours, feeding was discontinued and intravenous fluid was administered; 1/2 strength normal saline with 5% dextrose, 72 ml/kg per 24 hours (3 ml/kg per hour). Usually, 20 mmol/L of injection potassium chloride was added to 1 L of the infusion. Other treatments (antibiotics) received in the wards have been described elsewhere [14,15].

### Measurements

Case report forms were developed, pretested, and finalized for the acquisition of study-relevant data. The demographic information on admission such as age, sex, vaccinations, socioeconomic status, and lack of breastfeeding during the neonatal period were analyzed. Clinical features such as duration of diarrhea; vomiting, with duration; dehydration; weight for age; and weight for length/height z-score; reluctance to feed, with duration; fever, with duration; convulsion; oral thrush; hypoxemia; septic shock; hospital acquired infection; and outcome were also analyzed. The analyzed laboratory characteristics were hypoglycemia (random blood sugar <3.0 mmol/L), bacteremia (bacterial isolate from a single blood sample culture), hypokalemia (serum potassium < 3.5 mmol/L), hyperkalemia (serum potassium > 5.5 mmol/L), hyponatremia (serum sodium < 130.0 mmol/L) and hypernatremia (serum sodium >150.0 mmol/L), elevated serum creatinine (> 35.0 mmol/L in children <12 months old, <65.0 umol/L in children ≥12 months old), metabolic acidosis (serum TCO_2_ < 17.0 mmol/L), hypocalcemia (serum calcium < 2.12 mmol/L), and hypomagnesemia (serum magnesium < 0.70 mmol/L). Septic shock was defined as severe sepsis that was unresponsive to fluid resuscitation. Severe sepsis in diarrheal children has been defined in recent publications from Bangladesh [14,16]; originally adopted from the surviving sepsis guideline recommended by the American Pediatric Association [17,18], with minor modifications for children with diarrhea. Severe sepsis was defined as sepsis plus the presence of poor peripheral perfusion (weak or absent peripheral pulses), and a capillary refilling time greater than 3 seconds or age-specific hypotension. Sepsis was defined as the presence or presumed presence of infection with hyperthermia or hypothermia (rectal temperature >38∙5°C or <35∙0°C, respectively) and tachycardia in the absence of dehydration or after correction of dehydration.

### Procedure of identification of pathogens

Using the available aseptic precautions, the attending physician performed venipuncture and collected blood for culture; 10% povidone iodine followed by 70% rectified spirit was used to disinfect the puncture site, which was dried for 30 to 60 seconds. A standard pediatric blood culture bottle (BacT/Alert PF, Organon-Teknika, Durham, NC) was used to collect 2–5 mL of blood [19]. After the collection of blood, the microbiology laboratory, situated in the 2^nd^ floor of the hospital, immediately processed the bottles. Blood culture bottles were incubated in the automated BacT/Alert system. Initial positivity of specimens was flagged by the system and the flagged specimen was subsequently sub-cultured onto blood agar, MacConkey agar, and chocolate agar. Isolation and identification of pathogens were performed using standard bacteriologic procedure [20]. Antimicrobial susceptibility testing was performed with Mueller-Hinton agar plates, using a disc diffusion method.

### Analysis

All data were entered into SPSS for Windows (version 17.0; SPSS Inc., Chicago) and Epi-Info (version 6.0, USD, Stone Mountain, GA). Differences in proportion were compared by the Chi-square test. Student’s t-test was used to compare the means of normally distributed data and the Mann-Whitney test was used for the comparison of data that were not normally distributed. A p-value less than 0.05 was considered statistically significant. The strength of association was determined by calculating the odds ratio (OR) and its 95% confidence interval (CI). For the identification of risks for ileus in children having diarrhea with SAM, we initially analyzed the relevant variables in a univariate model (Table 1). Finally, for the adjustment of the covariates, we performed a multivariable logistic regression analysis (Table 2), where the dependent variable was ileus and the independent variables were those associated with ileus in the univariate model. We performed a second multivariable logistic regression analysis (Table 3) to evaluate the independent predictors of death, where death was the dependent variable and the variables associated with death in a univariate analysis acted as independent variables.

**Table 1:** Clinical and laboratory characteristics of children under five years old with diarrhea and severe acute malnutrition with (cases) and without ileus (controls).

Characteristic	Cases (n = 45)	Controls (n = 261)	OR	95% CI	p-value
**Male sex**	28 (62)	143 (55)	1.36	0.68–2.74	0.44
**Age in months (median, IQR)**	9.0 (5.0, 17.0)	9.7 (5.0, 18.0)	-	-	0.82
**Poor socio-economic condition**	37 (82)	222 (85)	0.81	0.33–2.05	0.79
**No EPI vaccination**	7 (16)	62 (24)	0.59	0.23–1.47	0.31
**Lack of intake of vitamin A**	20 (44)	146 (56)	0.63	0.32–1.25	0.21
**Non-breastfed from neonatal period**	8 (18)	42 (16)	1.13	0.45–2.75	0.95
**Duration of diarrhea (median, IQR)**	5.0 (3.0, 6.5)	4.0 (3.0, 5.0)	-	-	0.17
**Weight for length/height Z score (mean ± SD)**	-3.9 ± 1.7	-3.9 ± 1.5	0.04*	-0.43–0.52	0.86
**Weight for age Z score (mean ± SD)**	-5.5 ± 1.4	-5.1 ± 1.6	-0.45*	-0.95–0.05	0.08
**Vomiting**	10 (22)	41 (16)	1.53	0.65–3.53	0.39
**Duration of vomiting (median, IQR)**	2.0 (1.0, 4.3)	3.0 (2.0, 3.5)	-	-	0.26
**Reluctance to feed**	8 (18)	15 (6)	3.55	1.27–9.70	0.01
**Duration of reluctance to feed (median, IQR)**	5.0 (4.3,13.0)	10.0 (3.0, 15.0)	-	-	0.59
**Dehydration**	7 (16)	45 (17)	0.88	0.34–2.23	0.95
**Fever**	29 (64)	139 (53)	1.59	0.79–3.23	0.22
**Duration of fever (median, IQR)**	4.0 (3.0, 7.0)	4.0 (2.0, 5.0)	-	-	0.20
**Convulsion**	4 (9)	9 (3)	2.73	0.67–10.33	0.11
**Oral thrush**	7 (16)	50 (19)	0.78	0.30–1.95	0.72
**Hypoxemia**	5 (11)	25 (10)	1.18	0.37–3.50	0.79
**Septic shock**	9 (20)	17 (7)	3.59	1.36–9.34	<0.01
**Hospital acquired infection**	2 (4)	14 (5)	0.82	0.12–3.99	1.00
**Hypoglycemia on/after admission**	0 (0)	6 (2)	0.00	0.00–5.53	0.60
**Blood transfusion**	7 (16)	16 (6)	2.82	1.01–7.92	0.05
**Bacteremia**	7 (16)	23 (9)	1.91	0.69–5.10	0.18
**Hypokalemia**	25 (56)	94 (36)	2.22	1.12–4.42	0.02
**Hyperkalemia**	7 (16)	33 (13)	1.27	0.47–3.28	0.77
**Hyponatremia**	9 (20)	44 (17)	1.23	0.51–2.90	0.76
**Hypernatremia**	2 (4)	29 (11)	0.37	0.06–1.69	0.28
**Metabolic acidosis**	22 (49)	140 (54)	0.83	0.42–1.63	0.67
**Raised serum creatinine**	7 (16)	35 (13)	1.19	0.45–3.06	0.88
**Hypocalcemia**	20 (44)	67 (26)	2.32	1.15–4.66	0.02
**Hypomagnesemia**	2 (4)	9 (3)	1.30	0.35–4.50	0.67
**Outcome (Death)**	10 (22)	21 (8)	3.27	1.31–8.05	<0.01

Figures represent n (%), unless specified. OR: odds ratio. CI: confidence interval. IQR: inter-quartile range.

*: Mean difference

**Table 2:** Results of the logistic regression analysis revealing the independently associated factors with ileus in children below five years of age with severe acute malnutrition and diarrhea.

Characteristics	OR	95% CI	p-value
**Septic shock**	3.62	1.47–8.95	<0.01
**Reluctance to feed**	3.22	1.24–8.39	0.02
**Hypokalemia**	1.99	1.03–3.86	0.04
**Hypocalcemia**	1.67	0.82–3.41	0.16
**Blood transfusion**	1.73	0.32–4.26	0.81

OR: odds ratio. CI: confidence interval

**Table 3:** Results of logistic regression analysis identifying the independent predictors of death after adjusting for potential confounders in the study population.

Characteristics	OR	95% CI	p-value
**Septic shock**	168.84	19.27–1479.17	<0.01
**Ileus**	1.84	0.59–5.75	0.29
**Reluctance to feed**	0.95	0.15–6.11	0.96
**Hypokalemia**	1.64	0.61–4.42	0.33
**Hypocalcemia**	1.64	0.57–4.67	0.36
**Blood transfusion**	0.11	0.01–1.15	0.07

OR: odds ratio. CI: confidence interval

We also performed pathogen-specific data analysis and compared the differences in the proportion of bacterial pathogens between children with and without septic shock. We also compared the differences in the proportion between the overall and different bacterial pathogens, between children with and without ileus (Table 4). Only the Chi-square test was used for the comparison of the proportion.

**Table 4:** Association of ileus with bacterial pathogens from stool in children aged under five years with severe acute malnutrition and diarrhea.

Characteristic	Bacterial pathogens in children with ileus (n = 29)	Bacterial pathogens in children without ileus (n = 137)	OR	95% CI	p-value
**Total number of bacterial pathogens**	4 (14)	26 (19)	0.68	0.16–2.24	0.605
***Vibrio cholerae***	2 (7)	9 (7)	1.05	0.11–5.51	1.00
***Shigella***	1 (3)	12 (9)	0.37	0.08–2.73	0.469
**Non-typhoidal *Salmonella***	1 (3)	2 (1)	2.41	0.04–47.49	0.440
***Campylobacter* species**	0	1 (1)	0	0	1.00
**Mixed bacterial pathogens (*Shigella* and *Vibrio cholerae*)**	0	2 (1)	0	0–9.27	1.00

OR: odds ratio. CI: confidence interval

## Results

The review identified 45 cases and 261 controls. The cases, more often, were reluctant to feed, had septic shock, hypokalemia, and hypocalcemia on admission, and received blood transfusion during hospitalization, compared to the controls (Table 1).

In the logistic regression model, the dependent variable was ileus and the independent variables were septic shock, reluctance to feed, hypokalemia, hypocalcemia, and blood transfusion. In this model, ileus was independently associated with reluctance to feeding, septic shock, and hypokalemia (Table 2).

In a univariate analysis, the mortality rate was significantly higher in those with ileus compared to those without ileus (Table 1); however, in a multivariable logistic regression analysis, after adjusting for potential confounders such as septic shock, no association between ileus and death was noted (OR = 2.05, 95% CI = 0.68–6.14, p = 0.20). Moreover, in the multivariable logistic regression model shown in Table 3, death was the dependent variable and ileus, septic shock, reluctance to feed, hypokalemia, hypocalcemia, and blood transfusion were the independent variables. After adjusting for potential confounders, septic shock was identified as an independent predictor of death in our study population (Table 3).

Among the 26 children who had septic shock, 5 (19%) had bacteremia, and in the remaining 280 children without septic shock, 25 (9%) had bacteremia; however, the difference in bacteremia between the two groups was not statistically significant (p = 0.156). Stool culture was performed in 29 of 45 (64%) cases and 137 of 261(52%) controls (Table 4).

The proportion of the total number of bacterial pathogens isolated from stool was comparable between children with and without ileus (Table 4). In the pathogen-specific isolation, the proportion of *Vibrio cholera*, *Shigella*, Non-typhoidal *Salmonella*, *Campylobacter* species, and mixed bacterial pathogens (*Shigella* and *Vibrio cholerae*) was also comparable between the groups (Table 4).

## Discussion

To date, no study has attempted to identify the risk factors and outcome of ileus in children aged under five years with diarrhea and severe malnutrition. Some important observations were made in this study: first, children with SAM and diarrhea who developed ileus during hospitalization more often were reluctant to feed, had septic shock, and hypokalemia on admission; and second, these children had a higher mortality rate compared to those without ileus in a univariate analysis, however, after adjusting for potential confounders such as septic shock in a multivariable regression analysis, we observed no association between ileus and death. These observations need to be clarified for the formulation of better future interventions that may help to reduce deaths in children with diarrhea and SAM, especially in resource-poor settings.

The association of septic shock with ileus in children under five years old with diarrhea and severe malnutrition is understandable. Septic shock in children often stimulates oxidative stress, the endogenous production of nitric oxide [21], and the simultaneous surge of serum lactate [22–24]. This eventually results in abandoned vasodilatation followed by hypotension and reduced splanchnic circulation [25,26]. Absorption of food is often perceived to be greatly reduced in the gut because of compromised circulation in septic shock and, thus, contributes to the development of abdominal distension followed by ileus.

The association of hypokalemia with ileus in children having diarrhea and SAM is consistent with a number of previous studies [27,28]. The independent association of ileus with children being reluctant to feed is also understandable. Children with diarrhea and severe malnutrition who were reluctant to feed, more often presented with severe gut infection, leading to an edematous small bowel [18]. This phenomenon might contribute to the narrowing of the small bowel lumen and gaseous abdominal distension resulting in ileus.

The non-association between ileus and death in a multivariable regression analysis, despite an initial observation of higher deaths in a univariate analysis in children with diarrhea and SAM is critically important. Although a number of previous studies reported a high mortality in children with ileus but without diarrhea [7,29], ileus failed to predict deaths in this study population after adjusting for potential confounders (Table 3). In the regression analysis shown in Table 3, septic shock was found to be independently associated with death, underscoring the confounding effect of septic shock on the ability of ileus to predict death. Septic shock has been shown to be independently associated with death in a number of previous studies [30–32].

The lack of performance of viral isolation, including rotavirus, was one of the limitations of this study, although, the prevalence of rotavirus infection in SAM children is quite low due to the lack of receptors required for rotavirus attachment to villus cells in the intestine [13,33]. Potential misclassification bias in enrolling our study population during the chart analysis was another limitation of the study. Moreover, the retrospective nature of the study and the small sample size might have prevented the observation of an association between some of our variables of interest and ileus.

The current study; however, has several strengths. First, the data were abstracted from the electronic database of the largest diarrhea hospital in the world and during the data collection from different individuals at different time points, we did not find any inconsistency among the datasets. Second, this is the only study that has evaluated the risk factors and outcome of ileus in children with diarrhea and severe malnutrition. Third, there was scrupulous adherence to available standard treatment protocols for ileus in the ICU. Finally, the results of this study may be used in resource-limited settings where diarrhea and malnutrition are common.

In conclusion, our study noted that the deaths were significantly higher among children with diarrhea and severe malnutrition who had ileus, compared to those without ileus. Children below five years old who were hospitalized for diarrheal illness and presented with reluctance to feed, septic shock, and hypokalemia on admission, were at higher risk of developing ileus during hospitalization. Thus, the identification of these simple parameters in severely malnourished children with diarrhea on admission may prompt clinicians to be more vigilant in managing these conditions, especially in resource-limited settings in order to cure ileus and prevent further complications. A prospective study involving a larger sample of such children is crucial for the evaluation of our findings.
